# The epidemiology of alcohol consumption in Ethiopia: a systematic review and meta-analysis

**DOI:** 10.1186/s13011-019-0214-5

**Published:** 2019-06-11

**Authors:** Getinet Ayano, Kalkidan Yohannis, Mebratu Abraha, Bereket Duko

**Affiliations:** 1 Research and Training Department, Amanuel Mental Specialized Hospital, PO BOX: 1971 Addis Ababa, Ethiopia; 2 Department of Psychiatry, Dilla University, Dilla, Ethiopia; 3 Department of Psychiatry, Paulo’s Millennium Medical College, Addis Ababa, Ethiopia; 4 Department of Psychiatry, Hawassa University, Hawassa, Ethiopia

**Keywords:** Epidemiology, Alcohol consumption, Alcohol dependence, Ethiopia, Systematic review, Meta-analysis

## Abstract

**Background:**

Globally, excessive alcohol consumption is a major public health problem and is associated with social, mental, physical and legal consequences. However, no systematic review and meta-analysis has been performed to report the consolidated magnitude of alcohol consumption in Ethiopia.

**Methods:**

PubMed, EMBASE, and SCOPUS were systematically searched to identify pertinent studies. Subgroup and sensitivity analysis was conducted and Cochran’s Q- and the I^2^ test were used to assess heterogeneity. Publication bias was evaluated by using Egger’s test and visual inspection of the symmetry in funnel plots.

**Results:**

We included 26 articles with a total of 42,811 participants. The pooled current and lifetime prevalence of alcohol consumption was 23.86% (95%CI; 17.53–31.60) and 44.16% (95%CI; 34.20–54.62), respectively. The pooled prevalence of hazardous alcohol consumption was 8.94% (95%CI; 3.40–21.50). The prevalence of hazardous alcohol consumption was remarkably higher in men (11.58%) than in women (1.21%). The prevalence of current and lifetime alcohol consumptions among university students were 22.08% & 38.88% respectively. The pooled data revealed that male sex was found to be a significant predictor of hazardous alcohol consumption (OR 10.38; 95%CI 3.86 to 27.88) as well as current (OR 2.45; 95%CI 1.78 to 3.38) and lifetime (OR 2.14; 95%CI 1.39 to 3.29) consumption. The magnitude of alcohol consumption among university students was apparently lower than the magnitude in other population of the country. The current study suggested a remarkable recent increment in the magnitude of hazardous alcohol consumption in Ethiopia.

**Conclusion:**

The current study revealed that the prevalence of alcohol consumption in Ethiopia is comparable with the global estimates of alcohol consumption from the World Health Organization (WHO). The prevalence of hazardous alcohol consumption was remarkably higher in men (11.58%) than in women (1.21%). Male sex was found to be a significant predictor of alcohol consumption. The present study also suggested considerable recent increment in the magnitude of hazardous alcohol consumption in Ethiopia.

**Electronic supplementary material:**

The online version of this article (10.1186/s13011-019-0214-5) contains supplementary material, which is available to authorized users.

## Background

Globally excessive alcohol consumption is a significant public health problem and is responsible for about 6% of mortality and 5% of disability-adjusted life year’s (DALYs) lost worldwide [1]. The World Health Organization (WHO) estimate that, globally, about 53% of people aged 15 years and above have ever used alcohol and 39% used it in the last year [2]. A 2015 study found that around 4.9% of the world’s adult population is believed to suffer from alcohol use disorder [1]. According to scientific evidence in Africa, an estimated 43% of those aged 15 years or above have ever used alcohol and 30% used it in the last year [3]. The reported prevalence of alcohol use disorders (AUD) (defined by an Alcohol Use Disorders Identification Test (AUDIT) score ≥ 8) is estimated at 4% globally and 3% in Africa and is generally more prevalent among men [1].

In addition, a study conducted in South Africa (SA) revealed that 9% of the population aged 15 years or older engaged in risky or hazardous or harmful drinking. More men had hazardous drinking than women, 17 and 2.9% respectively [4]. Similarly, another hospital-based study in South Africa adults using Alcohol Use Disorder Identification Test (AUDIT) [5] found that 41.2% of men and 18.3% of women had hazardous drinking and 3.6% of men and 1.4% of women met criteria for probable alcohol dependence or harmful drinking as defined by AUDIT [6].

In Ethiopia, studies indicated that the prevalence of alcohol consumption has shown a significant increment, and generally hazardous drinking and alcohol dependence were more prevalent in men than in women [7–10]. In recent years, alcohol advertisements have become widespread in Sub-Saharan Africa (SSA) and in other regions of the world. Most advertisements propagate drinking as modern and associated with occupational and sexual achievements [11–13].

Epidemiologic evidence found that alcohol consumption has been linked with increased rates of pancreatitis [14, 15], liver cirrhosis [16], cardiovascular disease [17, 18], tuberculosis [19], mortality [20, 21], decrease productivity [22], disability [23], renal damage [24], lung cancer and diabetes [25, 26], crime [27], risky sexual behavior [28], unemployment [29, 30], poor academic performance [31, 32], stroke [33], and traffic fatalities [34, 35]. The medical and social costs of treating drinking and smoking-related illnesses are estimated to be in the billions [22].

However, to the best of our knowledge, no systematic review and meta-analysis has been performed to report the consolidated magnitude of alcohol use in Ethiopia. Therefore, the objective of this study was to conduct a systematic review and meta-analysis of studies conducted in Ethiopia on alcohol use and dependence, and to systematically summarize: (1) the prevalence of current and lifetime, as well as hazardous use alcohol use and dependence, (2) to estimate and compare the magnitude of alcohol consumption of between men and women, university students and other population as well as recent and past years and to formulate recommendations for future research as well as clinical practice.

## Methods

### Search process and study selection

An extensive search of relevant studies was conducted in three databases electronic databases (EMBASE, PubMed, and Scopus). We conduct our search in PubMed using the following terms and keywords: prevalence, epidemiology OR magnitude)) AND (alcohol OR substance OR alcohol drinking OR alcohol use OR abuse OR dependence OR hazardous drinking OR harmful use OR alcohol use disorder OR alcohol dependence OR substance use OR substance use disorder OR alcohol abuse OR alcohol dependence OR psychoactive substance OR psychoactive substance use) AND (factors OR risk factor OR risk OR determinant) AND Ethiopia. We looked at EMBASE and SCOPUS using database specific subject headings associated with the above keywords used in PubMed. The reference lists of eligible studies were also scanned to identify other pertinent to this review. We adhered PRISMA (Preferred Reporting Items for Systematic Reviews and Meta-Analyses) guidelines [36]. The identified studies were assessed by their titles, abstract, duplication as well as full-text contents against the predefined eligibility criteria,

### Eligibility criteria

Studies were included in the current systematic review and meta-analysis if they satisfy the following First, they were conducted using observational studies (cross-sectional and case-control study design); Second, measured the magnitude of alcohol consumptions (current, lifetime, and hazardous use as well as alcohol dependence). Thirdly, conducted in Ethiopia. Additionally, we excluded editorials, commentaries, reviews, conducted in nonhuman subjects and those not published in the English language.

### Methods for data extraction and quality assessment

Two reviewers (KY and MA) independently conducted data extraction from source documents. Disagreements were fixed by discussion and consensus. A prespecified form which was specifically designed to extract data of methodological and scientific quality was used. As recommended by PRISMA [37], the following data were extracted from each study: first authors name, source population, study design and setting, the gender of the participants, the sample size, year of publication, and the instrument used to measure alcohol consumption.

A modified version of NOS (the Newcastle-Ottawa Scale) [38] was utilized to appraise the quality of included in the meta-analysis studies. The instruments used to measure alcohol consumptions, statistical quality, sample representativeness, sample size and comparability between participants were the domains NOS scale uses to assess the quality of individual studies. We employed agreement beyond chance (unweighted kappa) for evaluation of the agreement levels between the two authors (KY and MA) during the quality assessment. The levels poor, slight, fair, moderate, substantial, and almost perfect levels of agreement were represented by the values 0, 01–0.02, 0.021–0.04, 0.041–0.06, 0.061–0.08, and 0.081–1.00, respectively [39].

### Definition of terms

Hazardous alcohol consumption or problematic alcohol use refers to the pattern of alcohol consumption that increases the risk of harmful consequences for the users or others [40]. In this review, hazardous alcohol consumption was considered when the studies assessed and reported the magnitude of hazardous alcohol consumption according to screening instruments used to estimate the level of problematic alcohol consumption such as CAGE (Cut down, Annoyed, Guilty, and Eye-opener), Alcohol, Smoking and Substance Involvement Screening Test (AUDIT), the Alcohol, Smoking, and Substance Involvement Screening Test (ASSIST). Thus, the hazardous alcohol consumption level represents the lifetime magnitude of problematic alcohol uses in Ethiopia.

### Data synthesis and analysis

We conducted the meta-analysis using a comprehensive meta-analysis software version 3. Random effect model was utilized to pool the overall prevalence of alcohol consumption [41]. We utilized the Q and the I^2^statistics to evaluate the evidence of heterogeneity between the studies included in the meta-analysis [41]. The values of 25, 50 and 75% represented a low, medium and high level of heterogeneity, respectively [42]. The level of significance was set at *P* < 0.05. We also performed subgroup and sensitivity analysis to determine the source of heterogeneity as well as to evaluate the prevalence across the groups. The presence of publication bias was evaluated by using Egger’s test and visual inspection of the symmetry in funnel plots.

## Results

### Identification of studies

**Fig. 1:**
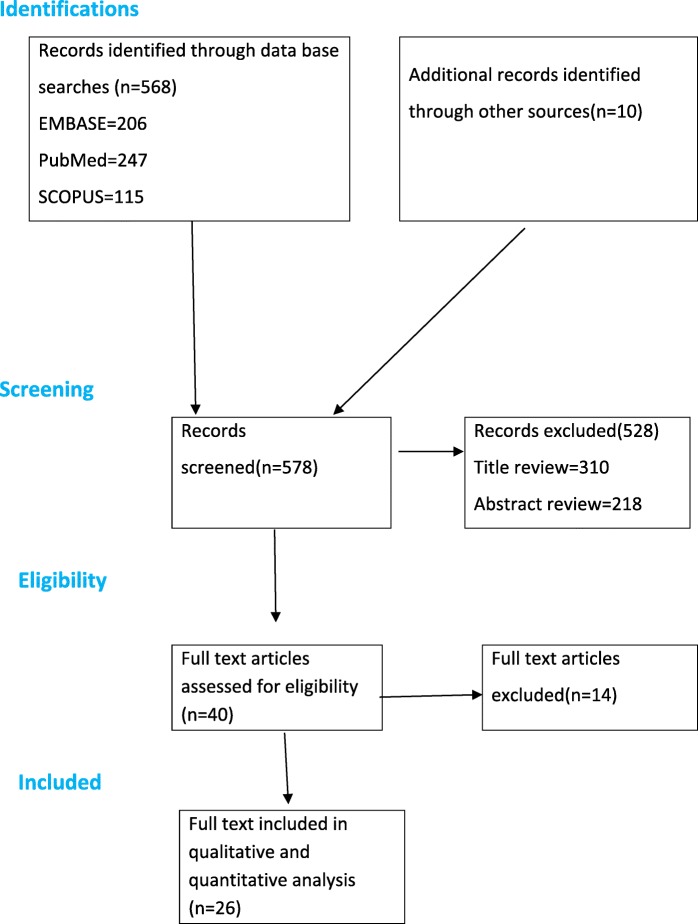
PRISMA flowchart of review search

Our electronic search resulted in 568 articles. Additionally, 10 relevant studies were identified through a manual search of the reference lists of the included articles. The review of the abstract and resulted in the exclusion of 528 studies as they did not meet the inclusion criteria **(**Fig. 1). We retrieved a full text of 40 for further screening and 14 of these were excluded.

### Characteristics of included studies

In this review, 26 relevant studies were included. The included studies were conducted between 1999 and 2017. From the total, 25 studies utilized a cross-sectional study design [7, 9, 43–65] and one study used case-control study design [66]. Five of the studies used community samples and twenty-one of the studies used samples from the institution. Standard diagnostic or screening instruments were used in 5 studies and self-report was used in 21 studies. Sixteen of the studies assessed current alcohol consumption, fifteen of the studies assessed lifetime alcohol consumption, five of the studies assessed hazardous consumptions, and only one of the studies assessed alcohol dependence (Table 1**).**

**Table 1:** Distribution of studies on alcohol use and dependence included in qualitative and quantitative analysis based on year, study design, sample size, instrument, country, response rate, study population and prevalence

Author (year) (reference number)	Study design (setting)	Sample size	Tool	Response rate	Residence	ALC use/hazardous	Study population, age	Prevalence
Dida N. et.al (2014) [39]	Cross sectional study (institution based)	603	Self-report	97.9	Oromia	Current USE	Any age, 15–29, students	Overall 23.6%(n/*N* = 142/603)
Alemseged F. et al. (2012) [40]	Cross sectional study (community based)	4352	Self-report	81.3	Oromia	Current Use	adults	Overall 7.1%(n/*N* = 307/4352) Men 8.5% (n/*N* = 177/2089 Women 5.7% n/*N* = 130/2263))
Tesfaye G,et al. (2013) [41]	Cross sectional study (institution-based study)	1022	Self-report	98.3%	Harar	Current Use	Any age, students	Overall 20%(n/*N* = 204/1022) Men 23.1%(n/*N* = 179/777) Women 10.5% n/*N* = 25/245)
						Lifetime	Any age, students	Overall 50.2%(n/*N* = 513/1022) Men53.8%(n/*N* = 418/777) Women 38.8% n/*N* = 95/245)
Hagos EG,et al. (2013) [42]	Cross sectional study (institution-based study)	271	Self-report	100%	Tigray	Ever use	Any age, students	Overall 25.1%(n/*N* = 68/271)
Mekonnen T. et.al (2017) [43]	Cross sectional study (institution)	725	CAGE, ASSIST	97.05%	South Ethiopia	Current use	Any age, students	Overall 24.7% (n/N = 179/725)
						Problematic use (CAGE)	Any age, students	Overall 10.2% (n/*N* = 74/725)
Alem A et.al (1999) [44]	Cross sectional study (comminity based)	10,468	85%	CAGE	South Ethiopia	Problematic drinking	Age above 15	Overall 3.7% (n/*N* = 385/10468 Men 7.5% (n/*N* = 329/4385 Women 0.9% n/*N* = 56/6083))
Tilahun M.et al. (2013) [45]	Cross sectional study (institution setting)	405	Self-report	Not reported	South Ethiopia	Current use	Youth	Overall 43.5%(n/*N* = 176/405)
Tadesse M (2014) [46]	Cross sectional study (institution setting)	611	Self-report	98%	South Ethiopia	Life time Use	Any age, students	Overall 64.7% (n/*N* = 395/611) Men 68.2(n/*N* = 296/434) Women 55.9% (n/*N* = 99/177)
Eshetu E.et al. (2006) [47]	Cross sectional study (institution setting)	600	Self-report	89%	Addis Ababa	Life time Use	Any age, students	Overall 56.7% (n/*N* = 312/550) Men 63.3%(n/*N* = 266/420) Women 35.4%(n/*N* = 46/130)
						Current use	Any age, students	Overall 31.1% (n/*N* = 171/550) Men 35.6%(n/*N* = 153/420) Women 13.8%(n/*N* = 18/130)
Alem A et.al (1999) [30]	Cross sectional study (institution setting)	10,203	CAGE	Not reported	Addis Ababa	Problem drinking (CAGE)	Age 15 and above	Overall 2.7%(n/*N* = 277/10203) Men 5.8%(n/*N* = 268/4597) Women 0.2% (n/N = 9/5606)
						Alcohol dependence	Age 15 and above	Overall 1 Men 1.9% Women 0.1%
Alemu H et al. (2007) [51]	Cross sectional study (Institution setting)	624	Self-report	Not reported	Amhara	Use	Youth	Overall 58%(n/*N* = 360/624)
Shiferaw D.et al. (2017) [50]	Cross sectional study (institution setting)	600	Self-report	92.6%	Somalia	Life time use	Any age, students	Overall 27.3%(n/*N* = 164/600) Men 35.6%(n/*N* = 133/396) Women 15.2%(n/N = 31/204)
						Current use	Any age, students	Overall 18.3%(n/*N* = 110/600)
Kassa A.et al. (2016) [53]	Cross sectional study (institution setting)	362	Self-report	94.5%	South Ethiopia	Current use	Any age, students	Overall 29.5%(n/*N* = 173/586) Men 30.3% (n/*N* = 148/479) Women 23.4%(n/N = 25/107)
						Life time use	Any age, students	Overall 48.7%(n/*N* = 285/586) Men 52.4%(n/*N* = 251/479) Women 31.8%(n/*N* = 34/107)
Reda AA. et.al (2012) [49]	Cross sectional study (institution setting)	1721	Self-report	91.1%	Harar	Current use	Any age, students	Overall 10.4%(n/N = 179/1721)
						Life time use	Any age, students	Overall 22.2%(n/*N* = 372/1721) Men 29.67%(n/*N* = 254/856) Women 13,78%(n/*N* = 118/851)
Gebreslassie M. et.al(2013) [52]	Cross sectional study (institution setting)	756	Self report	98.7%	Tigray	Current use	Any age, students	Overall 32.8%(n/*N* = 248/756) Men 41.9%(n/*N* = 186/444) Women 19.9%(n/*N* = 62/312)
						Life time use	`	Overall 34.52%(n/*N* = 261/756) Men 44.4%(n/*N* = 197/444) Women 20.5%(n/*N* = 64/312)
Malaju MT.et.al (2013) [62]	Unmatched Case control (institution setting)	405 (105 cases and 305 controls)	Self-report	98.8%	South Ethiopia	Current use	Youth	Overall 31.6%(n/*N* = 128/405)
Birhanu AM et.al (2011) [48]	Cross sectional study (institution setting)	651	Self-report	95.2%	Amhara	Life time use	Adolescents	Overall 59%(n/*N* = 384/651)
						Current use	Adolescents	Overall 40.9% (n/N = 266/651)
Deressa W. et.al (2010) [54]	Cross sectional study (institution setting)	622	Self-report	78%	Addis Ababa	Life time use	Any age, students	Overall 31.4%(n/*N* = 195/622) Men 35.4%(n/*N* = 151/426) Women 22.4%(n/*N* = 44/196)
						Current use	Any age, students	Overall 4.5%(n/N = 28/622) Men 5.6(n/N = 24/426) Women 2%(n/*N* = 4/196)
Tefrera S. et.al (2016) [28]	Cross sectional study (institution setting)	1500	AUDIT	Not reported	South Ethiopia	Hazardous drinking	Any age, students	Overall 21%(n/*N* = 309/1500) Men 31.8(n/*N* = 230/742) Women 10.4%(n/*N* = 79/758)
Mossie A. et.al (2011) [55]	Cross sectional study (community)	590	Self-report	90.8%	Jimma, Oromia	Current use	adults	Overall 34.4%(n/*N* = 203/590)
Gelaye B. et.al (2012) [56]	Cross sectional study (community)	2180	Self-report	Not reported	Ethiopia	Life time use	adults	Overall 77.3%(n/*N* = 1686/2180)
Fekadu A. et.al (2014) [57]	Cross sectional study (community)	1497	FAST	Not reported	Rural Ethiopia	Hazardous use	adults	Overall 22.6%(n/N = 312/1382)
Dessie y. et.al (2013) [58]	Cross sectional study (institution)	430	Self-report	97.3%	Oromia	Life time use	Any age, students	Overall 37.9%(n/*N* = 163/430)
Adere A. et.al (2017) [59]	Cross sectional study (institution)	655	Self-report	89.7%	Oromia	Life time use	Any age, students	Overall 33.1%(n/*N* = 217/655) Men 37.7%(n/N = 171/454) Women 22.9%(n/N = 46/201)
						Current use	Any age, students	Overall 27.9(n/*N* = 183/655) Men 31.5%(n/*N* = 143/454) Women 19.9%(n/*N* = 40/201)
Haile YG.et.al (2017) [60]	Cross sectional study (institution)	388	Self-report	91.9%	Amhara	Life time use	Any age, students	Overall 42.78%(n/*N* = 166/388)
Hersi L. et.al (2017) [61]	Cross sectional study (institution)	570	Self-report	95%	Oromia	Current use	Any age, students	Overall 42.78%(n/N = 166/388)

key: *CAGE* Cut down, Annoyed, Guilty, and Eye-opener, *ASSIST* Alcohol, Smoking and Substance Involvement Screening Test, *AUDIT* The Alcohol Use Disorders Identification Test, Student: University students

### The quality of the included studies

We used NOS (the Newcastle-Ottawa scale) with modifications to evaluate the quality of studies included in the meta-analysis. Our evaluation revealed that all the 26 studies were of good methodologic quality. The authors reach in conclusion that the study selection, measurement of outcomes, as well as the non-response bias were low. The agreed levels between the authors regarding the quality of the studies included the meta-analysis ranged from moderate to almost perfect levels (Kappa statistic 0.60–1). (Additional file 1: Table S1).

### The results of a pooled meta-analysis

#### Prevalence of current alcohol use

**Fig. 2:**
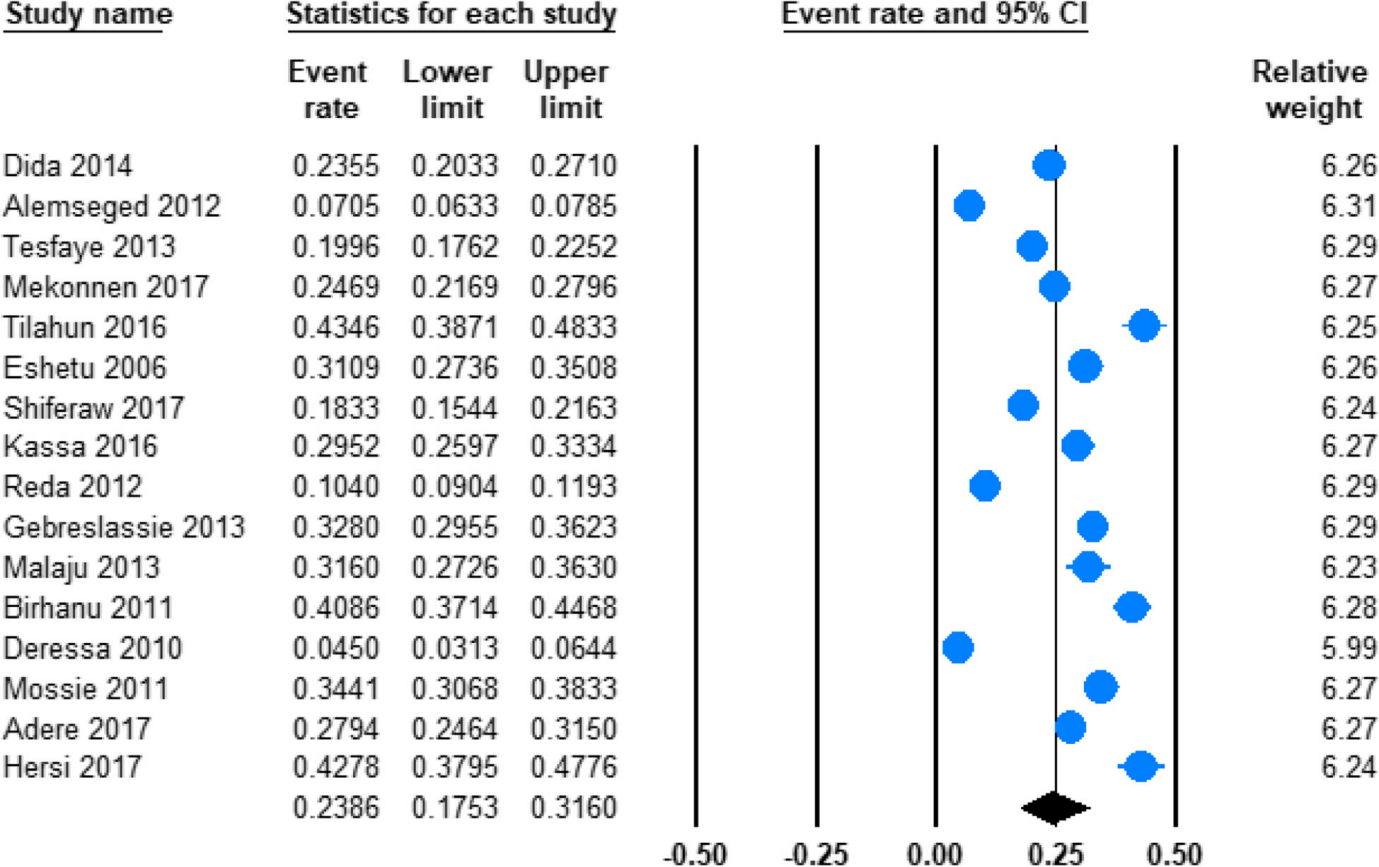
Forest plot of the prevalence of current alcohol use in Ethiopia

From the total, 16 studies measured the prevalence of current alcohol use in Ethiopia (Table 1). We used random effect model to combine these sixteen studies and provide the pooled estimates. The pooled prevalence of current alcohol use was found to be 23.86% (95% CI; 17.53–31.60) and the heterogeneity across the studies was significant (*I*^2^ = 98.76%; Q = 1205.05, df = 15, *p* < 0.001) (Fig. 2).

In our subgroup analysis, we found that the current prevalence of alcohol use was higher in men 22.06% (95%CI13.09–34.72) than in women 11.57% (95%CI 6.56–19.59). The reported heterogeneity was statistically significant for the prevalence estimates in men (*I*^2^ = 98.63; Q = 438.46, df = 6, *p* < 0.001) as well as women (*I*^2^ = 95.35; Q = 129.12, df = 6, *p* < 0.001). (See Table 2).

**Table 2:** Subgroup analysis of prevalence of alcohol use Ethiopia based on random effect analysis

Subgroup	Number of studies	Type of use	Estimates		Heterogeneity		
			Prevalence (%)	95% Confidence interval	I^2^(%)	Q (df)	*P* value
Sex							
Men	7	Current	22.06	13.09–34.72	98.63	438.46 (6)	*P* < 0001
Women	7	Current	11.57	6.56–19.59	95.35	129.12 (6)	*P* < 0001
Men	9	Life time	46.34	37.44–55.47	97.30	299.64 (8)	*P* < 0001
Women	9	Life time	25.02	13.91–40.79	97.50	319.68 (8)	*P* < 0001
Men	3	Hazardous	11.58	4.23–27.97	99.53	425.53 (2)	*P* < 0001
Women	3	Hazardous	1.21	1.005–6.61	99.29	283.01 (2)	*P* < 0001
Population type							
University Students	11	Current	22.08	16.67–28.65	97.56	409.35 (10)	*P* < 0001
Others (other population or community members)	5	Current	28.35	12.42–52.47	99.49	790.49 (4)	*P* < 0001
University Students	12	Life time	38.88	31.08–47.30	98.14	593.37 (11)	*P* < 0001
Others (other population or community members)	3	Life time	65.39	49.64–78.36	98.52	135.84 (2)	*P* < 0001
Year							
2014–2017 (recent years)	7	Current	29.31	23.19–36.38	95.09	122.27 (6)	*P* < 0001
Before 2014 (past years)	9	Current	20.09	12.08–31.51	99.17	958.59 (8)	*P* < 0001
2014–2017	6	Life time	42.10	31.45–53.54	97.56	204.84 (5)	*P* < 0001
Before 2014	9	Life time	45.54	31.14–60.72	99.39	1317.52 (8)	*P* < 0001
2014–2017	3	Hazardous	17.21	11.91–24.21	95.77	47.27 (2)	*P* < 0001
Before 2014	2	Hazardous	3.17	2.35–4.26	93.48	15.34 (1)	*P* < 0001

Key: Current use: use in the last 3 months; lifetime use: use at any time in their life: Hazardous: harmful use or abuse based on the standard instrument criteria

We also conducted a subgroup analysis of studies which provided information regarding the prevalence of current alcohol use among university students and other population members. The current prevalence of alcohol use was slightly higher among other population of the country 28.35% (95%CI 12.42–52.47) than among the university students 22.08% (95%CI 16.67–28.65). We found significant heterogeneity for studies conducted on the other population of the country (*I*^2^ = 99.49; Q = 790.49, df = 4, *p* < 0.001) as well as university students (*I*^2^ = 97.56; Q = 409.35; df = 10, *p* < 0.001). (See Table 2).

Moreover, in our stratified analysis by year, the current prevalence of alcohol use was significantly higher in recent (2014–2017) 29.31% (95%CI 23.19–36.38) than in past (before 2014) 20.09% (95%CI 12.08–31.51) years. The reported heterogeneity was considerable for the recent (*I*^2^ = 95.09; Q = 122.27, df = 6, *p* < 0.001) as well as past (*I*^2^ = 99.17; Q = 958.59; df = 8, *p* < 0.001) years. (See Table 2**).**

#### Prevalence of lifetime alcohol use

**Fig. 3:**
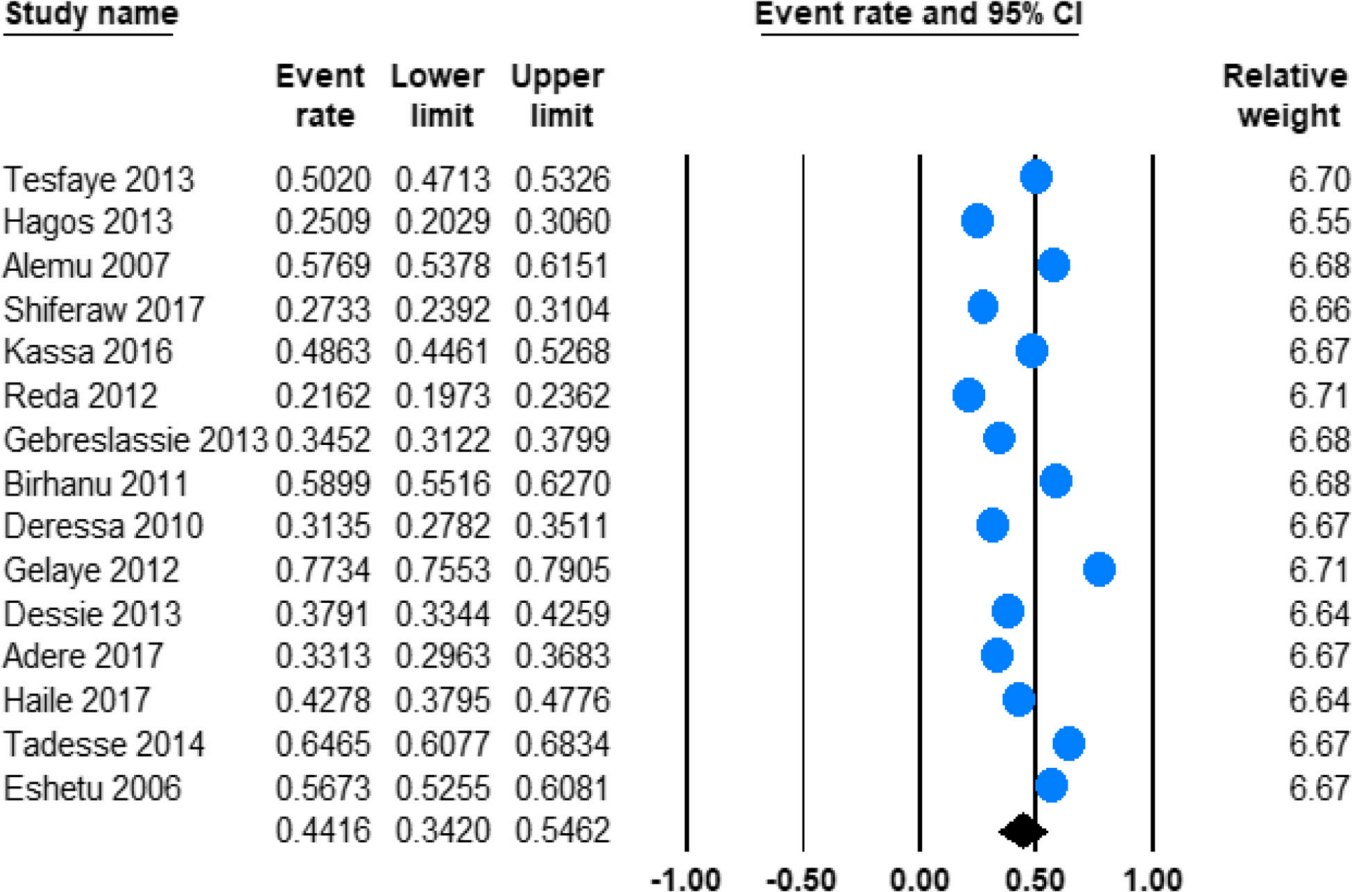
Forest plot of the prevalence lifetime alcohol use in Ethiopia

As illustrated in Tables 1, 15 studies estimated the prevalence of lifetime alcohol use in Ethiopia**.** Our meta-analysis found that the pooled prevalence of lifetime alcohol use was 44.16% (95% CI; 34.20–54.62) and the heterogeneity was considerable (*I*^2^ = 99.10%; Q = 1561.76, df = 14, *p* < 0.001). **(**Fig. 3**).**

In our subgroup analysis by gender, we found that the current prevalence of alcohol use was higher in men 46.34% (95%CI 37.44–55.47) than in women 25.02% (95%CI 13.91–40.79) with significant heterogeneity in both men (*I*^2^ = 97.30; Q = 299.64, df = 8, *p* < 0.001) and women (*I*^2^ = 97.50; Q = 319.68, df = 8, *p* < 0.001). (See Table 2).

Our meta-analysis also found a significantly higher prevalence of alcohol consumption among other population of the country 65.39% (95%CI 49.64–78.36) as compared with the prevalence among university students 38.88% (95%CI 31.08–47.30). A considerable heterogeneity was observed across the studies conducted among other population of the country (*I*^2^ = 98.52; Q = 135.84, df = 2, *p* < 0.001) as well as university students (*I*^2^ = 98.14; Q = 593.37; df = 11, *p* < 0.001). (See Table 2).

Moreover, in our stratified analysis the lifetime prevalence of alcohol use was almost similar in uses in recent (2014–2017) 42.10% (95%CI 31.45–53.54) and past (before 2014) 45.54% (95%CI 31.14–60.72) years with significant heterogeneity in both recent (*I*^2^ = 97.56; Q = 204.84, df = 5, *p* < 0.001) and past (*I*^2^ = 99.39; Q = 1317.52; df = 8, *p* < 0.001) years. (See Table 2.

#### Prevalence of hazardous alcohol use

**Fig. 4:**
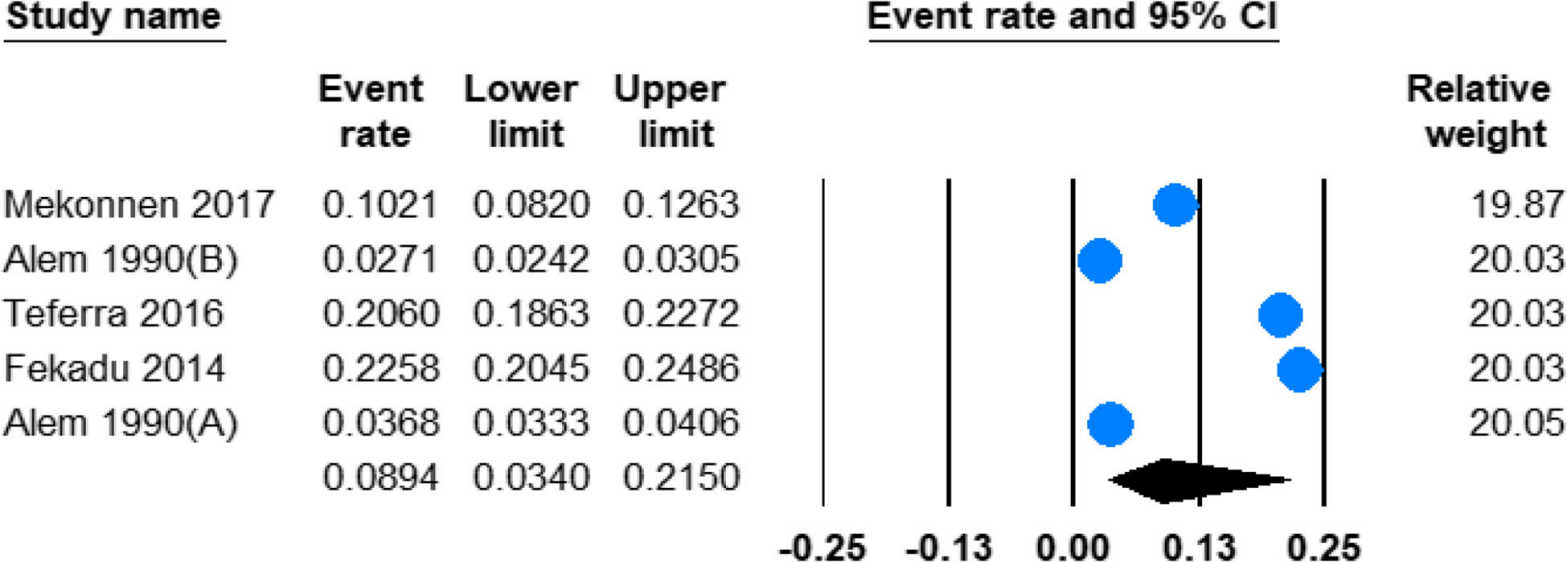
Forest plot of the prevalence of hazardous alcohol use in Ethiopia

Five studies provided information regarding the prevalence of hazardous alcohol use in Ethiopia **(**Table 1). The pooled prevalence of hazardous alcohol use was found to be 8.94% (95% CI;3.40–21.50) and the heterogeneity was significant (*I*^2^ = 99.68%; Q = 1249.95, df = 4, *p* < 0.001). (See Fig. 4).

The pooled prevalence of hazardous alcohol consumption was remarkably higher in men 11.58% (95%CI 4.23–27.97) than in women 1.21% (95%CI 1.005–6.61) with significant heterogeneity both in men (*I*^2^ = 99.53; Q = 425.53, df = 2, *p* < 0.001) and in women (*I*^2^ = 99.29; Q = 283.01, df = 2, *p* < 0.001). (See Table 2**).**

Furthermore, the prevalence of hazardous alcohol use was considerably higher in recent (2014–2017) 17.21% (95%CI 11.91–24.21) than in past (before 2014) 3.17% (95%CI 2.35–4.26) years. We identified significant heterogeneity in both recent (*I*^2^ = 95.77; Q = 47.27, df = 2, *p* < 0.001) and past (*I*^2^ = 93.48; Q = 15.34; df = 1, *p* < 0.001) years. (See Table 2).

#### The risk of being male and current alcohol use

**Fig. 5:**
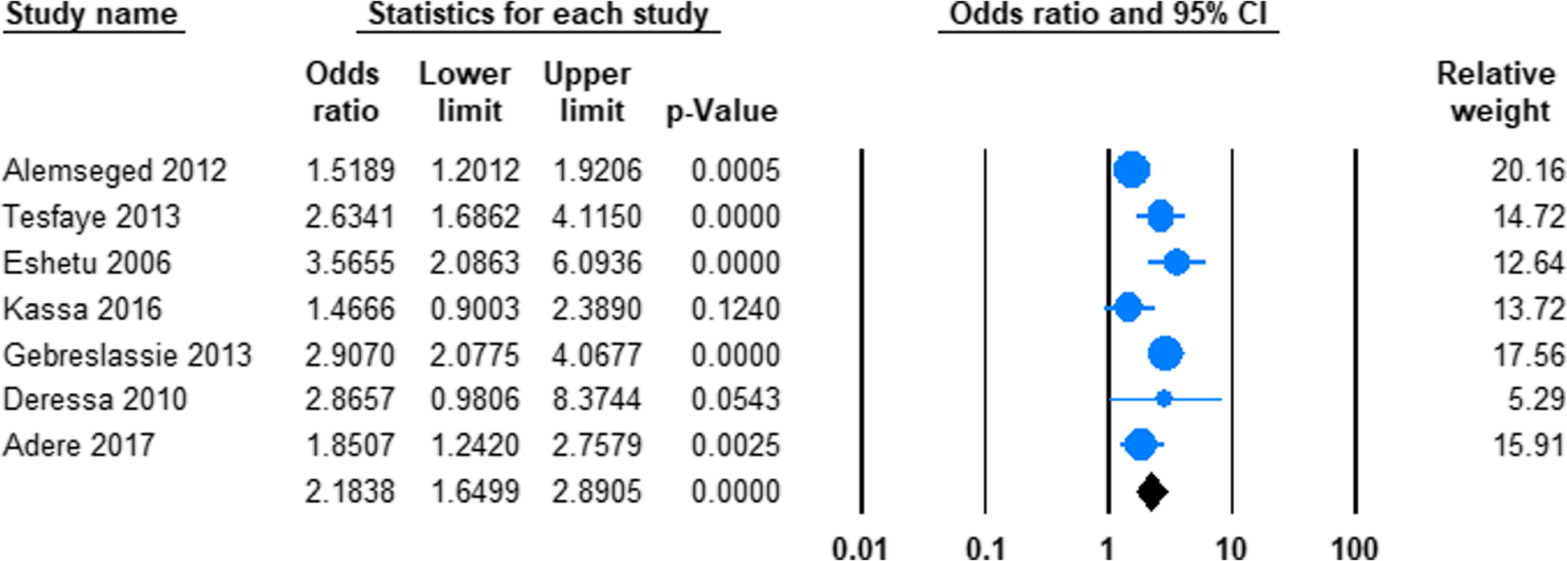
Forest plot of the risk of being male and current alcohol use in Ethiopia

Seven studies reported data on the risk of current alcohol use in men and women in Ethiopia **(**Table 1**).** The pooled odds ratio (OR) demonstrated that odds of current alcohol drinking were significantly higher in men than in women (OR 2.45; 95%CI 1.78 to 3.38, *P* < 0.001). (See Fig. 5).

#### The risk of being male and lifetime alcohol use

**Fig. 6:**
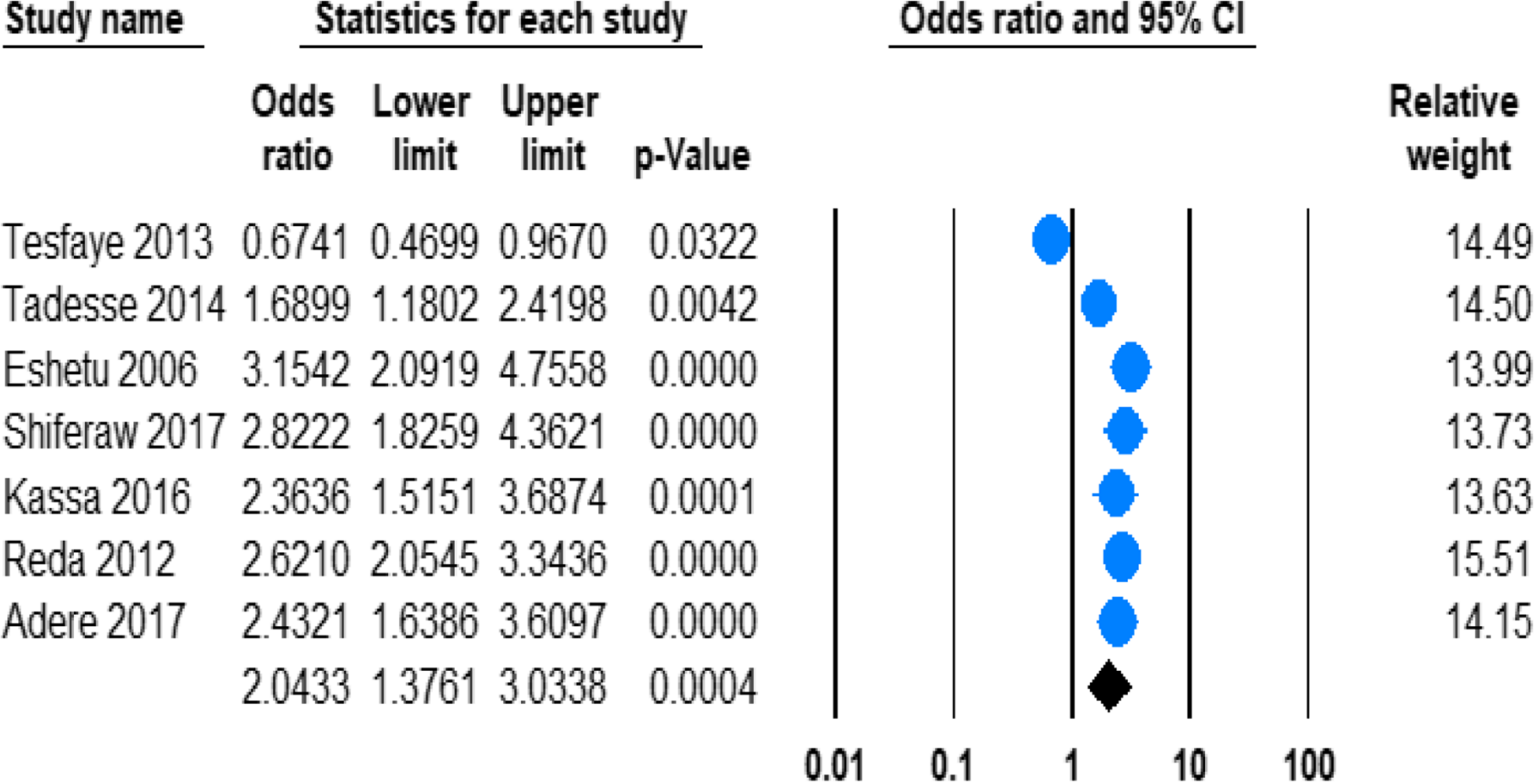
Forest plot of the being male and lifetime alcohol use in Ethiopia

Seven of the studies provided information regarding the risk of lifetime alcohol use in men and women in Ethiopia **(**Table 1**)**. The pooled odds ratio (OR) demonstrated that odds of lifetime alcohol drinking were significantly higher in men with than women (OR 2.14; 95%CI 1.39 to3.29, *P* = 0.0005). (See Fig. 6).

#### The risk of being male and hazardous alcohol use

**Fig. 7:**
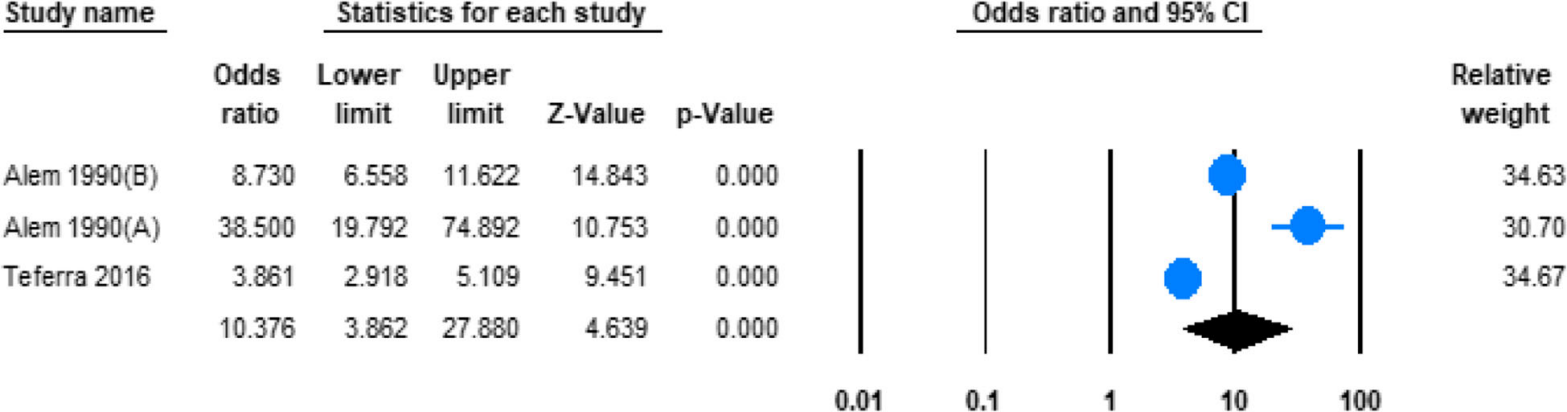
Forest plot of the risk of being male and hazardous alcohol use in Ethiopia

Three of the studies reported data on the risk of lifetime alcohol use in men and women in Ethiopia (Table 1). The pooled odds ratio (OR) demonstrated that odds of hazardous alcohol drinking were significantly higher in men with than in women (OR 10.38; 95%CI 3.86 to27.88, *P* < 0.0001). (See Fig. 7).

### Publication bias

**Fig. 8:**
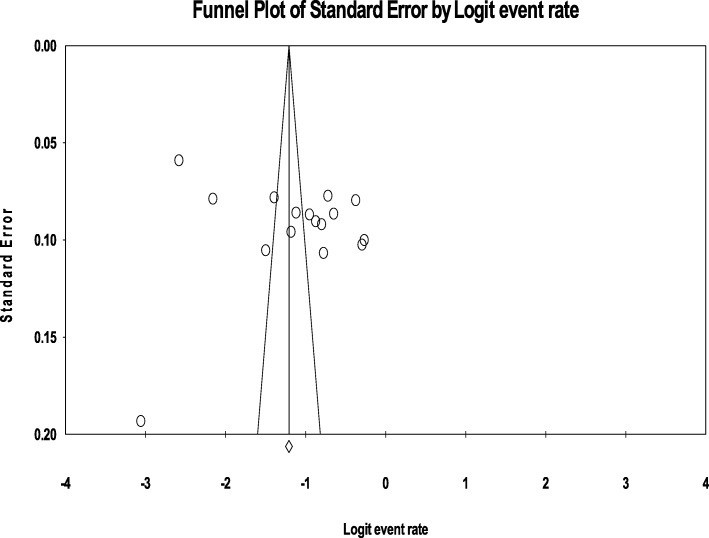
Funnel plot of publication bias for current alcohol use in Ethiopia

**Fig. 9:**
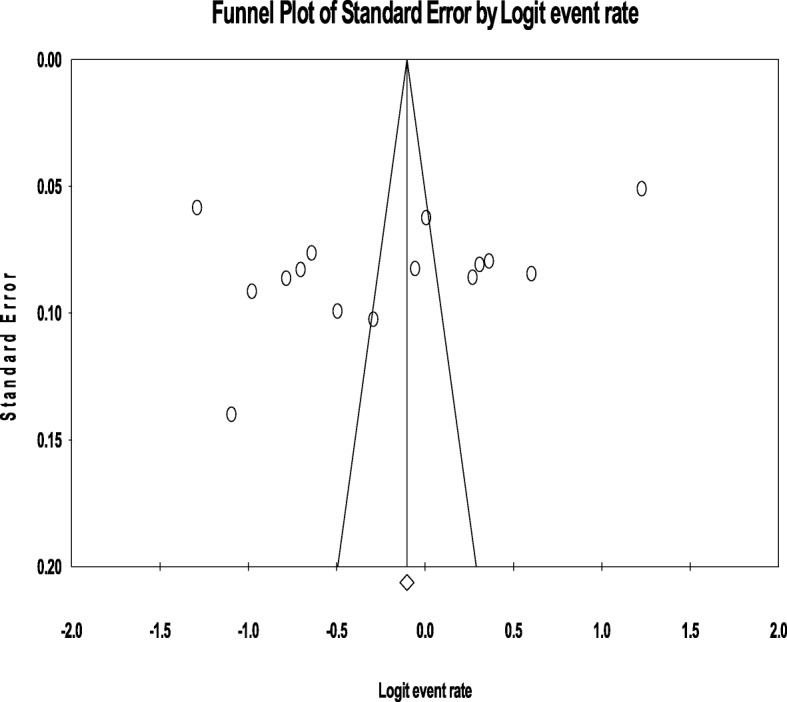
Funnel plot of publication bias for lifetime alcohol use in Ethiopia

No evidence of substantial publication bias was provided by the funnel plot and Egger’s regression tests for both the prevalence of current ((B = 12.59, SE = 10.69, *P* = 0.2584) as well as a lifetime (B = -16.28, SE = 11.43, *P* = 0.180) alcohol use in Ethiopia. (Figs. 8 and 9**)**.

### Sensitivity analysis

We also conducted a leave-one-out sensitivity analysis, for the purpose of further investigating the potential source of heterogeneity observed in the prevalence of current and lifetime alcohol use in Ethiopia. Our sensitivity analysis suggested that our findings were robust and not dependent on a single study. Our pooled estimated prevalence varied between 22.79% (14.48–30.48%) and 26.12% (20–40-34.25%) for the current and 41.60% (32.45–49.78%) and 46.05% (36.64–56.42%) for lifetime prevalence after deletion of a single study. (See Additional files 2 and 3**)**.

We also conducted a sensitivity analysis by restricting to the studies conducted after 2014 (recent) and before 2014 (past) years. We found a recent increment the prevalence of current alcohol consumption (29.31% vs. 20.09%) as well as hazardous alcohol consumption (17.21% vs. 3.68%) for this analysis. The observed difference in the magnitude of alcohol consumption in recent and past years was statistically significant for hazardous alcohol consumption (*P* < 0.0001) but not for current alcohol consumption (*P* = 0.151).

When conducting the analysis by restricting the analysis to studies conducted among students the prevalence of current and lifetime consumption was 22.08 and 38.88% respectively, as compared to the studies conducted in general population 28.35 and 65.39%. The observed difference was significant for the lifetime (*P* = 0.004) but not for current consumptions (0.546) consumption.

### Narrative review

#### Prevalence of alcohol dependence

We identified only one study that measured the prevalence of alcohol dependence in Ethiopia. The prevalence of alcohol dependence was 1%. The prevalence was significantly higher in men (1.9%) than women (0.1%).

## Discussion

### Main findings

To our knowledge, this is the first comprehensive systematic review and meta-analysis of the epidemiology of alcohol use disorders in Ethiopia which was conducted across 26 studies. The results of the meta-analysis revealed that the prevalence of alcohol consumption (including current, lifetime, and hazardous alcohol consumption) in Ethiopia was comparable with the global prevalence estimates of alcohol consumption from WHO reports [3]. This study also suggested that the pooled prevalence of hazardous alcohol consumption was remarkably higher in men (11.58%) than women (1.21%). Moreover, male sex was found to be a significant predictor of alcohol consumption. The present study also suggested considerable recent increment in the magnitude of alcohol consumption in Ethiopia.

In the current systematic review and meta-analysis, we observed that the existing scientific evidence on the epidemiology of alcohol consumption in Ethiopia was highly diverse by the gender of the participants, the type of alcohol consumption, the years of publications, type of population (student vs. other general population), and the locations of the studies. The studies reported the magnitude of alcohol consumption in participants from a different setting, and some studies reported the magnitude separately in males and females. Regarding the alcohol consumption patterns, some of the studies include current alcohol consumption, some of them included lifetime consumption, some studies reported hazardous consumptions and some of them included alcohol dependence.

In this study, the pooled prevalence of current alcohol use was found to be 23.86% (95% CI; 17.53–31.60)**.** Our finding was lower than studies done in China [67–69] and other western countries [70]. The variations might be explained by the possible psychological, the socioeconomic as well as cultural difference across the countries.

Regarding the lifetime alcohol consumptions, we found that the pooled prevalence estimates of lifetime alcohol use were 44.16% (95% CI; 34.20–54.62)**.** Our findings were in line with the findings from systematic review and meta-analysis done in sub-Saharan Africa which reported median prevalence of lifetime alcohol use 52% [71] as well as the reported prevalence in the reported global prevalence of alcohol consumption for the people age 15 and above (53%) by WHO [3].

As expected the pooled prevalence of current alcohol consumptions was apparently higher in men (22.06%) than in women (11.57%). Similarly, the study also suggested a remarkably higher rate of lifetime alcohol consumption in men (46.34%) than in women (25.02%). Additionally, this study revealed that males were 2.45 and 2.14 times more likely to be current and lifetime users of alcohol as compared with women. The sociocultural expectations and differences among males and females might be the possible reasons for the observed variation in the magnitude and risk of alcohol consumption between men and women. Nevertheless, the absolute reason for the variations needs further investigations. The findings of our meta-analysis are in agreement with the findings from Canada [72], the UK [73], and from meta-analysis findings in sub-Saharan African countries [71].

The pooled prevalence of hazardous alcohol consumption in Ethiopia (8.94%) was remarkably lower than the prevalence estimates in sub-Saharan Africa 15% [71]. In our stratified analysis, we found that the prevalence of hazardous alcohol use was considerably higher in men (11.58%) than women (1.21%). Additionally, we found that males were 10.38 times more likely to be hazardous drinkers as compared with women. In our narrative review, we found that the prevalence of alcohol dependence in Ethiopia was 1%. The prevalence was significantly higher in men (1.9%) than women (0.9%) [9]. The consequences of the higher drink in men and lower drink in women might be the possible reasons a significantly greater magnitude and risk of hazardous drinking as well as alcohol dependence among women than men [74, 75].

Finally, the prevalence of hazardous alcohol use was considerably higher in the recent (2014–2017) 17.21% (95%CI 11.91–24.21) than the past (before 2014) 3.17% (95%CI 2.35–4.26) years. This might be due to currently Ethiopia’s beverage industry is booming with increased foreign investment coupled with a significant increase in alcoholic beverage and industry by local investors and use of mass media advertisements to expose high proportions of large populations to messages through routine uses of existing media, such as television, radio, and newspapers in Ethiopia.

### The difference between the studies included in the present systematic review and meta-analysis

In the current study, the variation between the included studies resulted in a significant between-study heterogeneity in our meta-analysis for the current and lifetime alcohol consumption as well as hazardous alcohol consumption. To manage this heterogeneity and to make our findings meaningful, we used three main methods. Firstly, we used the appropriate model which control the effects of the observed heterogeneity during analysis. That means in this study we utilized a random effect model where the summary effect estimates are conservative than fixed effect models. Secondly, we conducted a leave one out sensitivity analysis and the results of our analysis revealed that the findings are robust and not dependent on a single study. Finally, we conducted subgroup and sensitivity analysis by sex of the participants, type of population (university students vs. general population), and years of study and we found that one of the main causes for the heterogeneity in our overall analysis was found to the variation in the magnitude of alcohol use in male and female participants. The years of the study was also found to be the other main cause for the heterogeneity for hazardous alcohol consumption. In addition, the main reasons for the significant heterogeneity between the studies for the lifetime alcohol consumption were found to be the lower magnitude of alcohol consumption in studies conducted among university students (38.88%) as compared to studies conducted in the general population (65.39%).

### Strengths and limitations

The current study has found a number of strengths: First, the use of predefined search strategy in order to reduce reviewer’s bias and conducting data extraction and quality evaluation by two independent reviewers to minimize the possible reviewer bias. Second, performing sensitivity and subgroup analysis based on type and patterns of alcohol consumption, type of population, the years of the study, and gender of the study participants. Thirdly, evaluating the alcohol consumption estimates across time is the other strength of the current study.

The limitations of the study include the small number of studies were used in our subgroup analysis particularly for hazardous consumption which reduces the precision of the estimate.

### Implications for future research and clinical practice

The current study identified some implication for the future research; our meta-analysis identified a recent increment in the magnitude of hazardous alcohol consumption which needs further investigation to assess the possible reasons for the remarkable recent increase as well as better ways of addressing the issues. We also identified sex difference in the magnitude of alcohol consumption which requires robust future studies to investigate the possible reasons for the variation. Finally, the concerned bodies need to give attention to address the problem including prevention and possible treatment strategies including strengthening of intergraded management of alcohol use disorders at primary healthcare level.

## Conclusion

This systematic review and meta-analysis revealed that nearly one out of five and two out of five of the population were current and lifetime alcohol users, respectively and roughly one in ten of the population were hazardous drinkers. The prevalence of alcohol consumption was remarkably high in males than in females and male sex was found to be a significant predictor of alcohol consumption. We also found that the magnitude of lifetime alcohol consumption was significantly low in university students than other population of the country but there is no significant difference between university students and the other population regarding current alcohol consumption. Additionally, the current study suggested a remarkable recent increment in the magnitude of hazardous alcohol consumption in Ethiopia.

Future epidemiologic studies focusing on the reasons for the recent increment in the magnitude of alcohol consumption as well as the possible reasons for the observed gender differences in the magnitude and risk of alcohol consumptions were warranted. Moreover, studies aiming at the incidence and determinates of alcohol consumptions among university students as well as other populations of the nation as well as studies focusing on the better ways for preventions and treatments of alcohol consumptions in Ethiopian context are recommended. Awareness tailored to specific genders, university students as well as other population is necessary. Finally, strengthening the integrated management of alcohol use disorders at primary health care level is warranted.

## Additional files

Additional file 1: **Table S1.** Summary of the quality and agreed level of bias and level of agreement on the methodological qualities of included studies in a meta-analysis based on sampling, outcome, response rate and method of analysis (DOCX 19 kb)

Additional file 2: Sensitivity analysis of prevalence for each study being removed at a time: prevalence and 95% confidence interval of current alcohol use in Ethiopia (DOCX 16 kb)

Additional file 3: Sensitivity analysis of prevalence for each study being removed at a time: prevalence and 95% confidence interval of lifetime alcohol use in Ethiopia (DOCX 16 kb)

## Data Availability

All data generated or analyzed during this study are included in this article.

## Abbreviations

ASSIST: Alcohol, Smoking, and Substance Involvement Screening Test
AUDIT: The Alcohol Use Disorders Identification Test, Student: University students
CAGE: Cut down, Annoyed, Guilty, and Eye-opener
CI: Confidence interval
OR: Odds ratio

## References

[1] Gowing LR, Ali RL, Allsop S, Marsden J, Turf EE, West R, Witton J (2015). Global statistics on addictive behaviours: 2014 status report. Addiction.

[2] WHO. Global status report on alcohol and health, 2014. Geneva: World Health Organization; 2014.

[3] Organization WH. Unit WHOMoSA: global status report on alcohol and health, 2014: World Health Organization; 2014.

[4] Peltzer K, Davids A, Njuho P. Alcohol use and problem drinking in South 600Africa: findings from a national population-based survey. Afr J Psychiatry. 2011;14(1):1–6. 10.4314/ajpsy.v14i1.65466 21509408

[5] Babor TF, Higgins-Biddle JC, Saunders JB, Monteiro MG, Organization WH (2001). AUDIT: the alcohol use disorders identification test: guidelines for use in primary health care.

[6] Pengpid S, Peltzer K (2011). Heever HVd: prevalence of alcohol use and associated factors in urban hospital outpatients in South Africa. Int J Environ Res Public Health.

[7] Teferra S, Medhin G, Selamu M, Bhana A, Hanlon C, Fekadu A (2016). Hazardous alcohol use and associated factors in a rural Ethiopian district: a cross-sectional community survey. BMC Public Health.

[8] Taffa N, Klepp K, Sundby J, Bjune G (2002). Psychosocial determinants of sexual activity and condom use intention among youth in Addis Ababa, Ethiopia. Int J STD AIDS.

[9] Kebede D, Alem A (1999). The epidemiology of alcohol dependence and problem drinking in Addis Ababa, Ethiopia. Acta Psychiatr Scand.

[10] Fekadu A, Alem A, Hanlon C (2007). Alcohol and drug abuse in Ethiopia: past, present and future. Afr J Drug Alcohol Stud.

[11] Bhana A (2008). Alcohol advertising, movies and adolescents. Addiction.

[12] Anderson P, De Bruijn A, Angus K, Gordon R, Hastings G (2009). Impact of alcohol advertising and media exposure on adolescent alcohol use: a systematic review of longitudinal studies. Alcohol Alcohol.

[13] Bryden A, Roberts B, McKee M, Petticrew M (2012). A systematic review of the influence on alcohol use of community level availability and marketing of alcohol. Health Place.

[14] Yen S, Hsieh C-C, MacMahon B (1982). Consumption of alcohol and tobacco and other risk factors for pancreatitis. Am J Epidemiol.

[15] Yadav D, Hawes RH, Brand RE, Anderson MA, Money ME, Banks PA, Bishop MD, Baillie J, Sherman S, DiSario J (2009). Alcohol consumption, cigarette smoking, and the risk of recurrent acute and chronic pancreatitis. Arch Intern Med.

[16] Corrao G, Bagnardi V, Zambon A, La Vecchia C (2004). A meta-analysis of alcohol consumption and the risk of 15 diseases. Prev Med.

[17] Rimm EB, Klatsky A, Grobbee D, Stampfer MJ (1996). Review of moderate alcohol consumption and reduced risk of coronary heart disease: is the effect due to beer, wine, or spirits?. Bmj.

[18] Ronksley PE, Brien SE, Turner BJ, Mukamal KJ, Ghali WA (2011). Association of alcohol consumption with selected cardiovascular disease outcomes: a systematic review and meta-analysis. Bmj.

[19] Lönnroth K, Williams BG, Stadlin S, Jaramillo E, Dye C (2008). Alcohol use as a risk factor for tuberculosis–a systematic review. BMC Public Health.

[20] Murray CJ, Lopez AD (1997). Global mortality, disability, and the contribution of risk factors: global burden of disease study. Lancet.

[21] Doll R, Peto R, Hall E, Wheatley K, Gray R (1994). Mortality in relation to consumption of alcohol: 13 years' observations on male British doctors. Bmj.

[22] Rehm J, Mathers C, Popova S, Thavorncharoensap M, Teerawattananon Y, Patra J (2009). Global burden of disease and injury and economic cost attributable to alcohol use and alcohol-use disorders. Lancet.

[23] Murray CJ, Lopez AD, Organization WH (1996). The global burden of disease: a comprehensive assessment of mortality and disability from diseases, injuries, and risk factors in 1990 and projected to 2020: summary.

[24] Perneger TV, Whelton PK, Puddey IB, Klag MJ (1999). Risk of end-stage renal disease associated with alcohol consumption. Am J Epidemiol.

[25] Freudenheim JL, Ritz J, Smith-Warner SA, Albanes D, Bandera EV, Van Den Brandt PA, Colditz G, Feskanich D, Goldbohm RA, Harnack L (2005). Alcohol consumption and risk of lung cancer: a pooled analysis of cohort studies. Am J Clin Nutr.

[26] Rimm EB, Chan J, Stampfer MJ, Colditz GA, Willett WC (1995). Prospective study of cigarette smoking, alcohol use, and the risk of diabetes in men. Bmj.

[27] Haggård-Grann U, Hallqvist J, Långström N, Möller J (2006). The role of alcohol and drugs in triggering criminal violence: a case-crossover study. Addiction.

[28] Halpern-Felsher BL, Millstein SG, Ellen JM (1996). Relationship of alcohol use and risky sexual behavior: a review and analysis of findings. J Adolesc Health.

[29] Luoto R, Poikolainen K, Uutela A (1998). Unemployment, sociodemographic background and consumption of alcohol before and during the economic recession of the 1990s in Finland. Int J Epidemiol.

[30] Crawford A, Plant MA, Kreitman N, Latcham RW (1987). Unemployment and drinking behaviour: some data from a general population survey of alcohol use. Addiction.

[31] Bergen HA, Martin G, Roeger L, Allison S (2005). Perceived academic performance and alcohol, tobacco and marijuana use: longitudinal relationships in young community adolescents. Addict Behav.

[32] Cox RG, Zhang L, Johnson WD, Bender DR (2007). Academic performance and substance use: findings from a state survey of public high school students. J Sch Health.

[33] Reynolds K, Lewis B, Nolen JDL, Kinney GL, Sathya B, He J (2003). Alcohol consumption and risk of stroke: a meta-analysis. Jama.

[34] Donovan DM, Marlatt GA, Salzberg PM (1983). Drinking behavior, personality factors and high-risk driving. A review and theoretical formulation. J Stud Alcohol.

[35] Híjar M, Carrillo C, Flores M, Anaya R, Lopez V (2000). Risk factors in highway traffic accidents: a case control study. Accid Anal Prev.

[36] Reviews UoYCf, Dissemination: Systematic reviews: CRD's guidance for undertaking reviews in health care: University of York, Centre for Reviews & Dissemination; 2009.

[37] Moher D, Shamseer L, Clarke M, Ghersi D, Liberati A, Petticrew M, Shekelle P, Stewart LA (2015). Group P-P: preferred reporting items for systematic review and meta-analysis protocols (PRISMA-P) 2015 statement. Syst Rev.

[38] Stang A (2010). Critical evaluation of the Newcastle-Ottawa scale for the assessment of the quality of nonrandomized studies in meta-analyses. Eur J Epidemiol.

[39] Landis J. Richard, Koch Gary G. (1977). The Measurement of Observer Agreement for Categorical Data. Biometrics.

[40] Babor T, Higgins-Biddle J, Saunders J, Monteiro M (2007). The alcohol use disorders identification test: guidelines for use in primary care. 2001.

[41] Borenstein M, Hedges LV, Higgins J, Rothstein HR (2010). A basic introduction to fixed-effect and random-effects models for meta-analysis. Res Synth Methods.

[42] Higgins JP, Thompson SG, Deeks JJ, Altman DG (2003). Measuring inconsistency in meta-analyses. BMJ.

[43] Dida Nagasa, Kassa Yibeltal, Sirak Teshome, Zerga Ephrem, Dessalegn Tariku (2014). Substance use and associated factors among preparatory school students in Bale Zone, Oromia Regional State, Southeast Ethiopia. Harm Reduction Journal.

[44] Alemseged F, Haileamlak A, Tegegn A, Tessema F, Woldemichael K, Asefa M, Mamo Y, Tamiru S, Abebe G (2012). Risk factors for chronic non-communicable diseases at gilgel gibe field research center, Southwest Ethiopia: population based study. Ethiop J Health Sci.

[45] Tesfaye Gezahegn, Derese Andualem, Hambisa Mitiku Teshome (2014). Substance Use and Associated Factors among University Students in Ethiopia: A Cross-Sectional Study. Journal of Addiction.

[46] Hagos EG, Asfeha GG, Berihu BA (2016). Prevalence of substance abuse among regular degree health science students in Sheba University College in Mekelle Town, Tigray-Ethiopia. J Neurosci Rural Pract.

[47] Mekonen Tesfa, Fekadu Wubalem, Mekonnen Tefera Chane, Workie Shimelash Bitew (2017). Substance Use as a Strong Predictor of Poor Academic Achievement among University Students. Psychiatry Journal.

[48] Alem A, Kebede D, Kullgren G (1999). The epidemiology of problem drinking in Butajira, Ethiopia. Acta Psychiatr Scand.

[49] Tilahun M, Ayele G (2013). Factors associated with age at first sexual initiation among youths in Gamo Gofa, south West Ethiopia: a cross sectional study. BMC Public Health.

[50] Tadesse M (2014). Substance abuse and sexual HIV-risk behaviour among Dilla University students, Ethiopia. Educ Res.

[51] Eshetu E, Gedif T (2006). Prevalence of khat, cigarette and alcohol use among students of technology and pharmacy, Addis Ababa University. Ethiop Pharm J.

[52] Birhanu AM, Bisetegn TA, Woldeyohannes SM (2014). High prevalence of substance use and associated factors among high school adolescents in Woreta town, Northwest Ethiopia: multi-domain factor analysis. BMC Public Health.

[53] Reda AA, Moges A, Wondmagegn BY, Biadgilign S (2012). Alcohol drinking patterns among high school students in Ethiopia: a cross-sectional study. BMC Public Health.

[54] Shiferaw D, Kinati T, Fufa G, Assefa L. Prevalence rate of alcohol use and its associated factors among undergraduate students of Jigjiga University. Global J Addict Rehab Med. 2017;4(2):1–6.

[55] Alemu H, Mariam DH, Belay KA, Davey G (2007). Factors predisposing out-of-school youths to HIV/AIDS-related risky sexual behaviour in Northwest Ethiopia. J Health Popul Nutr.

[56] Gebreslassie M, Feleke A, Melese T (2013). Psychoactive substances use and associated factors among Axum university students, Axum town, North Ethiopia. BMC Public Health.

[57] Kassa A, Wakgari N, Taddesse F (2016). Determinants of alcohol use and khat chewing among Hawassa University students, Ethiopia: a cross sectional study. Afr Health Sci.

[58] Deressa W, Azazh A (2011). Substance use and its predictors among undergraduate medical students of Addis Ababa University in Ethiopia. BMC Public Health.

[59] Mossie Andualem, Kindu Dagmawi, Negash Alemayehu (2016). Prevalence and Severity of Depression and Its Association with Substance Use in Jimma Town, Southwest Ethiopia. Depression Research and Treatment.

[60] Gelaye B, Lemma S, Deyassa N, Bahretibeb Y, Tesfaye M, Berhane Y, Williams MA (2012). Prevalence and correlates of mental distress among working adults in Ethiopia. *Clinical practice and epidemiology in mental health*. CP & EMH.

[61] Fekadu A, Medhin G, Selamu M, Hailemariam M, Alem A, Giorgis TW, Breuer E, Lund C, Prince M, Hanlon C (2014). Population level mental distress in rural Ethiopia. BMC Psychiatry.

[62] Dessie Y, Ebrahim J, Awoke T. Mental distress among university students in Ethiopia: a cross sectional survey. Pan Afr Med J. 2013;15(1):1–8. 10.11604/pamj.2013.15.95.2173 PMC3810159 24198889

[63] Adere A, Yimer NB, Kumsa H, Liben ML (2017). Determinants of psychoactive substances use among Woldia University students in northeastern Ethiopia. BMC Res Notes.

[64] Haile YG, Alemu SM, Habtewold TD (2017). Common mental disorder and its association with academic performance among Debre Berhan University students, Ethiopia. Int J Ment Heal Syst.

[65] Ereg D, Gesesew H, Tesfay K, Hersi L, Tesfaye M, Krahl W (2017). Mental distress and associated factors among undergraduate students at the University of Hargeisa, Somaliland: a cross-sectional study. Int J Ment Heal Syst.

[66] Malaju MT, Asale GA (2013). Association of Khat and alcohol use with HIV infection and age at first sexual initiation among youths visiting HIV testing and counseling centers in Gamo-Gofa zone, south West Ethiopia. BMC Int Health Hum Rights.

[67] Wei H, Derson Y, Shuiyuan X, Lingjiang L, Yalin Z (1999). Alcohol consumption and alcohol-related problems: Chinese experience from six area samples, 1994. Addiction.

[68] Millwood IY, Li L, Smith M, Guo Y, Yang L, Bian Z, Lewington S, Whitlock G, Sherliker P, Collins R (2013). Alcohol consumption in 0.5 million people from 10 diverse regions of China: prevalence, patterns and socio-demographic and health-related correlates. Int J Epidemiol.

[69] HAO WEI, YOUNG DERSON, LI LINGJIANG, XIAO SHUIYUAN (1998). Psychoactive substance use in three sites in China: Gender differences and related factors. Psychiatry and Clinical Neurosciences.

[70] Edwards G (1994). Organization ROfEWH: alcohol policy and the public good.

[71] Francis JM, Grosskurth H, Changalucha J, Kapiga SH, Weiss HA (2014). Systematic review and meta-analysis: prevalence of alcohol use among young people in eastern Africa. Tropical Med Int Health.

[72] Brenner DR, Haig TR, Poirier AE, Akawung A, Friedenreich CM, Robson PJ (2017). Alcohol consumption and low-risk drinking guidelines among adults: a cross-sectional analysis from Alberta's tomorrow project. Health Promot Chronic Dis Prev Can.

[73] Ely M, Wadsworth MEJ, Longford NT, Hardy R (1999). Gender differences in the relationship between alcohol consumption and drink problems are largely accounted for by body water. Alcohol Alcohol.

[74] Smart RG, Adlaf EM, Knoke D (1991). Use of the CAGE scale in a population survey of drinking. J Stud Alcohol.

[75] Chan AW, Pristach EA, Welte JW (1994). Detection by the CAGE of alcoholism or heavy drinking in primary care outpatients and the general population. J Subst Abus.

