# Supplementary material for: The epidemiology of alcohol consumption in Ethiopia: a systematic review and meta-analysis
Source: Subst Abuse Treat Prev Policy. 2019 Jun 11;14:26. doi: 10.1186/s13011-019-0214-5 (PMC6558840; doi:10.1186/s13011-019-0214-5)
Supplement: Supplementary file 1 — Table S1. Summary of the quality and agreed level of bias and level of agreement on the methodological qualities of included studies in a meta-analysis based on sampling, outcome, response rate and method of analysis (DOCX 19 kb) [file 13011_2019_214_MOESM1_ESM.docx]

Additional file 1: **Table S1** Summary of the quality and agreed level of bias and level of agreement on the methodological qualities of included studies in a meta-analysis based on sampling, outcome, response rate and method of analysis

| Study | Overall agreement and precision | | | Nos quality (score on 0 to 9 scale) |
| --- | --- | --- | --- | --- |
|  | Percentage of agreement | Kappa value | Level of agreement |  |
| Dida (2014) | 75 | 0.60 | Moderate | 7 |
| Tesfaye (2013) | 100 | 1 | Almost perfect | 8 |
| Hagos (2013) | 75 | 0.60 | Moderate | 7 |
| Mekonen (2017) | 100 | 1 | Almost perfect | 8 |
| Tadesse (2014) | 100 | 1 | Almost perfect | 9 |
| Eshetu et.al. (2006) | 100 | 1 | Almost perfect | 8 |
| Shiferaw (2017) (42) | 75 | 0.60 | Moderate | 7 |
| Kassa (2016) | 100 | 1 | Almost perfect | 9 |
| Gebreslassie (2013) | 100 | 1 | Almost perfect | 9 |
| Deressa (2010) | 100 | 1 | Almost perfect | 9 |
| Dessie (2013) | 75 | 0.60 | Moderate | 7 |
| Adere (2017) | 100 | 1 | Almost perfect | 8 |
| Reda AA et.al (2012) [9] | 100 | 1 | Almost perfect | 8 |
| Mekoneni et.al (2017) | 100 | 1 | Almost perfect | 8 |
| Alem et.al. (1990) | 100 | 1 | Almost perfect | 9 |
| Tefera et.al. et al. (2067) | 100 | 1 | Almost perfect | 8 |
| Fekadu et al. (2014) | 100 | 1 | Almost perfect | 8 |
| Birhanu et al. (2011) | 100 | 1 | Almost perfect | 8 |
| Alem et al. (1990) | 100 | 1 | Almost perfect | 8 |
| Haile et al. (2017) | 100 | 1 | Almost perfect | 9 |
| Gelaye (2012) | 100 | 1 | Almost perfect | 8 |
| Hersi et.al. (2015)   \|  \| \| --- \| | 100 | 1 | Almost perfect | 9 |
| Malaju et.al (2009 | 100 | 1 | Almost perfect | 9 |
| Mossie et.al (2013) | 100 | 1 | Almost perfect | 9 |
| Alemseged.et. al (2012) | 100 | 1 | Almost perfect | 9 |
