# Supplementary material for: The epidemiology of alcohol consumption in Ethiopia: a systematic review and meta-analysis
Source: Subst Abuse Treat Prev Policy. 2019 Jun 11;14:26. doi: 10.1186/s13011-019-0214-5 (PMC6558840; doi:10.1186/s13011-019-0214-5)
Supplement: Supplementary file 2 — Sensitivity analysis of prevalence for each study being removed at a time: prevalence and 95% confidence interval of current alcohol use in Ethiopia (DOCX 16 kb) [file 13011_2019_214_MOESM2_ESM.docx]

Additional file 2: Sensitivity analysis of prevalence for each study being removed at a time: prevalence and 95% confidence interval of current alcohol use in Ethiopia

| Study excluded | prevalence | 95%CI |
| --- | --- | --- |
| Dida N. et.al(2014) | 23.88 | 17.19-32.16 |
| Alemseged F. et. al.(2012) | 25.72 | 20.40-31.86 |
| Tesfaye G,et. al(2013) | 24.13 | 17.32-32.57 |
| Mekonnen T.et.al(2017) | 23.80 | 17.09-32.12 |
| Tilahun e.et. al(2016) | 22.79 | 16.58-30.48 |
| Eshetu E.et. al(2006) | 23.42 | 16.87-31.55 |
| Shiferaw .et. al(2017) | 24.26 | 17.54-32.54 |
| Kassa.et. al(2016) | 23.15 | 16.91-31.69 |
| Reda.et. al(2011) | 25.10 | 14.48-32.12 |
| Gebreslassie M.et.al(2013) | 23.32 | 16.77-31.46 |
| Malaqu et.al (2013) | 23.39 | 16.88-31.46 |
| Birhanu AM et.al(2011) | 22.91 | 16.66-30.65 |
| Deressa W.et.al(2010) | 26.12 | 19.35-34.25 |
| Mossie A.et.al(2011) | 23.24 | 16.79-31.28 |
| Adere A.et.al(2017) | 23.60 | 16.96-31.84 |
| Hersi .et.al(2017) | 22.83 | 16.59-30.55 |

Key. The analysis is based on random effect model
