# Supplementary material for: The epidemiology of alcohol consumption in Ethiopia: a systematic review and meta-analysis
Source: Subst Abuse Treat Prev Policy. 2019 Jun 11;14:26. doi: 10.1186/s13011-019-0214-5 (PMC6558840; doi:10.1186/s13011-019-0214-5)
Supplement: Supplementary file 3 — Sensitivity analysis of prevalence for each study being removed at a time: prevalence and 95% confidence interval of lifetime alcohol use in Ethiopia (DOCX 16 kb) [file 13011_2019_214_MOESM3_ESM.docx]

Additional file 3: Sensitivity analysis of prevalence for each study being removed at a time: prevalence and 95% confidence interval of lifetime alcohol use in Ethiopia

| Study excluded | prevalence | 95%CI |
| --- | --- | --- |
| Tesfaye G,et. al(2013) | 43.73 | 32.91-55.19 |
| Hagos et.al (2013) | 45.65 | 35.28-56.42 |
| Alemu et.al (2017) | 43.20 | 32.74-54.31 |
| Shiferaw .et. al(2017) | 45.47 | 35.10-56.26 |
| Kassa.et. al(2016) | 43.84 | 33.24-55.04 |
| Reda.et. al(2011) | 46.05 | 36.64-55.75 |
| Gebreslassie M.et.al(2013) | 44.88 | 34.29-55.95 |
| Birhanu AM et.al(2011) | 43.11 | 32.67-54.20 |
| Deressa W.et.al(2010) | 45.13 | 34.65-56.06 |
| Gelaye et.al (2012) | 41.60 | 33.86-49.78 |
| Desse .et.al(2013) | 44.62 | 34.09-55.65 |
| Adere A.et.al(2017) | 44.99 | 34.46-55.99 |
| Haile .et.al(2017) | 44.26 | 33.74-55.32 |
| Tadesse et. al(2014) | 42.69 | 32.45-53.60 |
| Eshetu ,et. al(2003) | 43.27 | 32.81-54.37 |

Key. The analysis is based on random effect model
